# NUAK: never underestimate a kinase

**DOI:** 10.1042/EBC20240005

**Published:** 2024-11-18

**Authors:** George L. Skalka, Declan Whyte, Dominika Lubawska, Daniel J. Murphy

**Affiliations:** 1 School of Cancer Sciences, University of Glasgow, Glasgow, U.K.; 2 CRUK Scotland Institute, Garscube Estate, Glasgow G61 1BD, U.K.

**Keywords:** AMPK-related kinase, ARK5, NUAK1, NUAK2, Phosphatase regulation, SNARK

## Abstract

NUAK1 and NUAK2 belong to a family of kinases related to the catalytic α-subunits of the AMP-activated protein kinase (AMPK) complexes. Despite canonical activation by the tumour suppressor kinase LKB1, both NUAKs exhibit a spectrum of activities that favour tumour development and progression. Here, we review similarities in structure and function of the NUAKs, their regulation at gene, transcript and protein level, and discuss their phosphorylation of specific downstream targets in the context of the signal transduction pathways and biological activities regulated by each or both NUAKs.

## Introduction

NUAK1 (aka ARK5, OMPHK1) and NUAK2 (aka SNARK, OMPHK2) comprise one branch of a broader family of Serine/Threonine kinases defined by amino acid sequence homology to the kinase domains of the catalytic α-subunits of the AMP-activated protein kinases, collectively called the AMPK-related kinases or ARKs [1–4]. Despite canonical activation by the upstream kinase and well-established tumour suppressor, LKB1 (aka STK11), NUAKs are implicated in a number of roles more commonly associated with tumour development and cancer progression [5–7]. For instance, NUAK1 has been shown to play key roles in cancer cell survival during energetic or oxidative stress [8,9], while NUAK2 is frequently amplified in a spectrum of human cancers [5] and both participate in facilitating cell motility required for cancer cell dissemination and metastasis [10]. Growing interest in targeting NUAKs in cancer has yielded a range of small molecule inhibitors, reviewed in considerable detail recently [11]; however, judicious use of such inhibitors requires a deeper understanding of NUAK biology. Here, we review similarities and differences in structure and function of the NUAKs and discuss their more established roles in signal transduction and biological function, particularly in relation to their roles in cancer.

## Gene, transcript and protein structure

**Figure 1:**
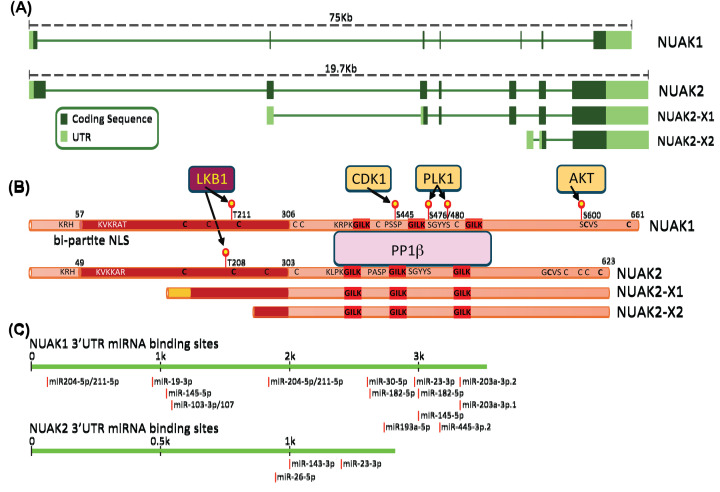
Structural alignment of NUAK1 and NUAK2 (**A**) Schematic of primary transcripts encoded by *NUAK1* and *NUAK2* including introns and UTRs. (**B**) Alignment of NUAK1 and NUAK2 proteins. Experimentally validated phosphosites and upstream kinases are indicated, along with the positions of GILK motifs, nuclear localisation sequences, and approximate location of cysteine residues (conserved cysteines in bold print). Alignment of putative NUAK2-X1 and NUAK2-X2 isoforms is shown. (**C**) Alignment of miRNAs targeting the *NUAK1* or *NUAK2* 3′UTR.

The gene encoding human NUAK1 resides at q23.3 of Chromosome 12 and comprises 6 short exons followed by a long 7th exon that includes an extensive 3′ untranslated region (3′UTR), with the entire primary transcript spanning over 75 kb. Human *NUAK2* resides at q32.1 of Chromosome 1 in a region that is frequently amplified in several cancers, including mammary, hepatic, pulmonary, uterine and ovarian cancers, along with melanoma [5]. The structure of the *NUAK2* gene closely mirrors that of *NUAK1*, although with significantly shorter intronic sequences, the major *NUAK2* primary transcript spans just under 20 kb (Figure 1A). The NUAK proteins share 58% amino acid identity along their entire length. Sequence identity is strongest between their protein kinase domains, which additionally show strong similarity to the kinase domains of the AMP-activated protein kinase (AMPK) α-subunits, encoded by *PRKAA1* and *PRKAA2*, and to the extended AMPK-related family of kinases [1]. Central to this kinase domain is the activation loop, which is conserved across all AMPK-related kinases (ARKs) and contains the threonine target for upstream kinases, phosphorylation of which is required for NUAK kinase activity. Similar to AMPK and other ARKs, NUAKs are canonically activated by LKB1 [1]; however, in LKB1-deficient cancer cells, NUAK activity is maintained by an as-yet undefined mechanism [12]. Unique amongst the ARKs, both NUAKs contain three GILK motifs enabling direct binding to the catalytic subunit of the protein phosphatase PP1β, enabling regulation of PP1β via phosphorylation of its regulatory subunits (see section on NUAK substrates, below) [10]. Both NUAKs also share a bi-partite nuclear localisation sequence (NLS) spanning the N-terminus of the kinase domain, functionally validated for NUAK1 [13]. A second putative NLS flanking the first GILK motif of NUAK1 is partially conserved in NUAK2 but has not been experimentally validated in either kinase (Figure 1B). Interestingly, NCBI RefSeq predicts two additional transcripts encoding N-terminally truncated isoforms of NUAK2: a 496 amino acid isoform X1 and a 381 amino acid isoform X2. Isoform X1 lacks the first 170 amino acids of full-length NUAK2 and thus contains a truncated kinase domain which lacks the bi-partite NLS but does contain the activation loop. Isoform X2 lacks the first 247 amino acids, including most of the kinase domain and the activation loop, but retains the GILK motifs and the entire C-terminal regulatory domain and should therefore retain PP1β binding. Although neither isoform has been experimentally validated to date, it is possible that X2 may function as a competitive inhibitor of NUAK activity towards PP1β, whereas X1 may be limited to cytosolic roles. Additionally, novel NUAK1 transcripts were recently detected in specific embryonic brain cell types using long-read single-cell RNA-Sequencing [14].

## Transcriptional regulation of NUAKs

Both NUAKs are widely expressed in normal human tissue with transcript detection by RNA-SEQ in all major tissues, except for bone marrow where NUAK1 expression is negligible [15]. NUAK1 shows highest expression in brain and lowest expression in pancreatic, spleenic and gastrointestinal tissues, while NUAK2 is poorly expressed in the adult brain and most highly expressed in gastrointestinal tissues, kidney, spleen and thyroid, with some suggestion that adult tissues may selectively favour expression of one or the other NUAK [15]. Analysis of developing murine embryos showed widespread expression of *Nuak1 (Omphk1)* in neuroectoderm and developing epidermal tissues, whereas embryonic expression of *Nuak2 (Omphk2)* is restricted to the neuroectoderm, with strongest expression in the developing forebrain and midbrain regions [16]. Genetic disruption of *Nuak1* results in failure to close the ventral body wall, termed Omphalocoele, and perinatal lethality in mice, while disruption of both *Nuaks* prevents closure of the neural tube and is embryonic lethal [16,17]. Accordingly, aberrant expression or mutation of NUAKs is linked to a spectrum of neurological abnormalities that have been reviewed extensively recently [18].

Physiological regulation of NUAK expression is poorly understood, however, expression of NUAKs is widespread across a spectrum of human cancers [11] and the factors that regulate NUAK expression in cancer are starting to emerge. Elevated expression of NUAK1 in Multiple Myeloma was shown to be induced by members of the large MAF transcription factor family, which are homologous to the avian retroviral oncogene *v-Maf*, and two functional MAF response elements were identified in the *NUAK1* promoter region [19]. More recently, expression of both NUAKs was found to be induced by TGFβ in a variety of mammalian cell types, including normal human fibroblasts [20]. Induction of NUAK2 expression by TGFβ is mediated by direct binding of SMAD2/3 complexes to an enhancer located in the first intron of the *NUAK2* gene. Although induction of both NUAKs was found to require kinase activity of TGFβR1 (aka ALK5) and expression of SMAD4, direct transcriptional regulation of *NUAK1* by SMAD2/3 was not established. In the same study, treatment with inhibitors of MEK or p38 MAPK also suppressed TGFβ-induced expression of NUAK proteins.

Two groups independently reported regulation of NUAK2 expression by the HIPPO pathway [21,22]. The HIPPO pathway comprises a regulatory kinase cascade that culminates in activation of the Large Tumour Suppressor protein kinases, LATS1 and LATS2, which inhibit the transcriptional co-activators, Yes-associated protein (YAP1) and TAZ, encoded by *WWTR1*. Phosphorylation of YAP1 by LATS results in 14-3-3-mediated cytosolic sequestration and/or protein degradation. Disruption of the HIPPO kinase module is common in several cancers, including hepatocellular carcinoma (HCC) and Mesothelioma, amongst others, resulting in nuclear accumulation of YAP1 and TAZ. Nuclear YAP1/TAZ in turn activate gene expression upon binding TEAD family transcription factors [23–25]. In a LATS-refractory model of HCC driven by expression of YAP1^S127A^, chromatin immuno-precipitation of TEAD4-bound DNA identified *NUAK2* as a candidate HIPPO-regulated gene. Both YAP1 and TEAD were found to bind to *NUAK2* super-enhancer regions in cholangiocarcinoma and mesothelioma cell lines, and acute overexpression of YAP1 upregulated *NUAK2* mRNA and protein, while depletion of YAP1 or both YAP1 & TAZ each reduced *NUAK2* expression [22]. Consistent results were independently found in MDA-MB231 breast cancer cells, wherein YAP, TAZ and TEAD were again shown to bind to *NUAK2* enhancer sequences and to positively regulate *NUAK2* mRNA expression [21]. Interestingly, the latter study also identified functional AP1 binding sites in the same enhancer region, and expression of a dominant negative JUN reduced FBS-induced *NUAK2* expression [21], potentially explaining the impact of MEK/MAPK inhibitors on TGFβ-induced NUAK2 protein levels [20]. Expression of NUAK1 in fibroblasts is similarly dependent on YAP1/TAZ/TEAD [26].

## Translational and post-translational regulation of NUAKs

Translation of NUAKs, particularly NUAK1, is regulated by several microRNAs (miRs; Figure 1C). The vast majority are reportedly linked to NUAK1’s roles in cancer cell migration, epithelial to mesenchymal transition and/or metastasis, e.g., miR203 [27,28], miR204 [29,30], and miR211 [31]. Of note, miR211 was additionally shown to regulate NUAK1 during neuronal differentiation [32]. Others, such as miR96 [33], miR145 [34] and miR143 [35,36] link NUAK1 or NUAK2 to more general roles in cancer. Additionally, a number of long non-coding (LNC) or circular RNAs are reported to promote NUAK expression by counteracting miRs that inhibit NUAK translation, resulting in tumour promotion/progression. These include LINC00958 in nasopharyngeal cancer [37], FGD5-AS1 and Circ_0000033 in breast cancer [38,39], NEAT in non-small cell lung cancer, LINC00922, Circ_0003998 and HOTAIR in HCC [39–41].

NUAK kinase activity is canonically activated upon phosphorylation of T211 on NUAK1, and T208 on NUAK2, by the upstream kinase LKB1. Mutation of these threonines results in complete inactivation of kinase activity [1]. According to the PhophoSitePlus post-translational modification resource (PP-PTMR) [42], a number of other sites on each NUAK have been found to be phosphorylated, for instance in large-scale phosphoproteomic studies [43,44]; however, detailed experimental evidence of phosphorylation is lacking in the majority of instances. At least 2 studies have independently reported phosphorylation of NUAK1^S600^ by AKT [45,46]. Although an S600A mutant retained full kinase activity *in vitro*, only a single peptide substrate was tested and context-dependent regulation of NUAK1 activity following S600 phosphorylation cannot be ruled out [47]. Interestingly, this site sits adjacent to an RxCV**S**xD/EN motif that is conserved in both NUAKs and is predicted by the PP-PTMR to be phosphorylated by several ARKs, in particular by the MARK family of AMPK-related kinases [42], suggesting potential crosstalk between other ARKs and the NUAKs.

The cell cycle regulator Polo-like kinase (PLK1) was shown to phosphorylate NUAK1 on S476 and S480, triggering βTRCP-dependent NUAK1 degradation during late G2/M [48]. Phosphorylation of NUAK1 by PLK1 required prior phosphorylation of NUAK1 on S445 by Cyclin-dependent kinase 1 (CDK1). Activating phosphorylation of PLK1 was in turn shown to be regulated by NUAK1-dependent inhibition of PP1β^MYPT1^, and treatment with NUAK inhibitors blocked cell cycle progression of U2OS osteosarcoma cells [49]. A similar effect on cell cycle progression was observed in MiaPaCa-2 pancreatic cancer cells, wherein NUAK1 was found to regulate PLK4-dependent centrosome duplication in S phase [50]. PLK1 plays a key role in promoting centrosome maturation during mitosis and later centriole disengagement [51]. Consistent with NUAK being a major regulator of PLK1, endogenously expressed NUAK1 localises to the centrosomes during mitosis [50].

The canonical upstream activator of NUAKs, LKB1, is frequently lost in a spectrum of cancer types, including pulmonary, pancreatic, and cervical cancers, amongst others [52]. Both NUAKs nonetheless retain kinase activity in LKB1-deficient cancers cells, such as HeLa and A549 cells. Indeed, wild type, but not T211A mutant, Flag-tagged NUAK1 was shown to be phosphorylated and active when overexpressed in HeLa cells [12]. NUAK2 also retains activity in HeLa cells, evidenced by residual phosphorylation of the common NUAK target, MYPT1, in cells treated with the highly-selective NUAK1 inhibitor, HTH-01-015. RNAi-mediated depletion of NUAK2 ablated this residual phosphorylation [12]. The upstream kinase responsible for NUAK T211/T208 phosphorylation in the absence of LKB1 has yet to be identified. The PP-PTMR [42] predicts that CAMKK1 or CAMKK2 may substitute for LKB; however, treatment of HeLa cells with the potent CAMKK inhibitor STO-609 had no impact on NUAK activity, as measured by phosphorylation of MYPT1, despite complete inhibition of AMPK^T172^ phosphorylation [12]. On the other hand, calcium ionophore treatment did increase phosphorylation of both NUAK1^T211^ and MYPT1^S445^, whereas treatment of cells with the calcium chelator, BAPTA, or with a PKC inhibitor, Gö6976, each reduced NUAK activity. Depletion of PKCα was found to reduce expression of both NUAKs independently of proteasome activity, accounting for the observed changes in NUAK activity; however, the NUAK activation loop does not conform to a PKC consensus motif (R/KxSxR/K) and thus the T211/T208 site is unlikely to be a direct substrate for PKC. Additionally, neither Gö6976 nor PKCα depletion affected NUAK1 mRNA levels and so their effects on protein expression are post-transcriptional and may reflect regulation of NUAK translation. Interestingly, in U2OS (LKB1 wild-type) cells, NUAK1 inhibitor treatment specifically reduced activating phosphorylation of AMPKα^T172^ in response to calcium ionophore treatment but not in response to phenformin or the AMPK agonist A769662, whereas in HeLa cells (LKB1 null), AMPKα^T172^ phosphorylation in response to all three stimuli was reduced by NUAK1 inhibition. CAMKK2 is a known activator of AMPK and is the main kinase responsible for AMPKα^T172^ phosphorylation in LKB1-deficient cells [53]. These data thus suggest that NUAK1 may be an activator of CAMKK, upstream of AMPK [8,12]. Consistent with this hypothesis, CAMKK^S445^ resides within a consensus AMPK/ARK motif and is predicted by the PP-PTMR to be phosphorylated by NUAK1, although this has yet to be verified experimentally.

**Figure 2:**
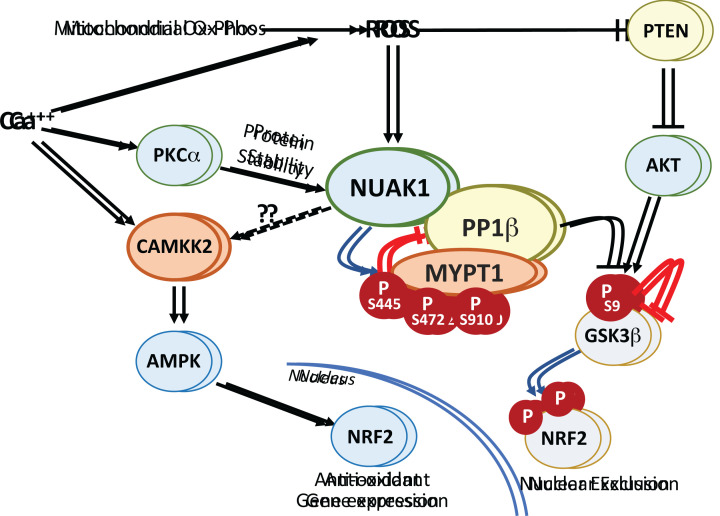
Regulation of NUAK1 by Calcium and Reactive Oxygen Species (ROS) Activation of NUAK1 attenuates cytosolic PP1β complexes via phosphorylation of the regulatory MYPT1 subunit. This activity is required to enable efficient inhibition of GSK3β by AKT signalling downstream of ROS inactivation of PTEN, facilitating nuclear translocation of the anti-oxidant master transcription factor NRF2 (encoded by *NFE2L2*). Calcium and ROS also activate AMPK via CAMKK2. CAMKK2 contains a consensus ARK phosphosite that may be targeted by NUAK1.

Beyond phosphorylation, NUAK1 activity is also increased by oxidative stress, similar to AMPK [9,54,55]. Highly reactive oxygen species (ROS) produced in the mitochondria are rapidly converted to less reactive hydrogen peroxide, which can react with the thiol group of cysteine, modulating protein conformation and/or activity [56]. NUAK1 contains 9 cysteine residues, 5 of which are conserved in NUAK2, including one in the invariant ARK activation loop and another, 5 amino acids from the C-terminal end of both proteins (Figure 1B). Using iodoacetamide to specifically label reduced thiol groups, mass spectrometric analysis showed oxidation of all 9 NUAK1 cysteines following treatment of U2OS cells with hydrogen peroxide, which correlated with rapidly increased phosphorylation of MYPT1 [9]. Consistently, NUAK1 was found to be required for nuclear translocation of the anti-oxidant transcription factor NRF2, which it promotes by attenuating PP1β^MYPT1^-dependent re-activation of GSK3β [9]. In the absence of NUAK1, GSK3β phosphorylates NRF2 preventing its accumulation in the nucleus [57]. Oxidative stress was subsequently found to drive NUAK1 eviction from the nucleus to accumulate in the cytosol, where it can counteract GSK3β−mediated suppression of NRF2 translocation [58]. Moreover, elevated NFκB activity was found to protect NUAK1-deficient ovarian cancer cells from ROS-induced cell death [59]. Interestingly, activation of AMPK by ROS was shown to be dependent on CAMKK2 rather than LKB1 and independent of changes in the AMP:ATP ratio [54]. Direct activation of NUAK by ROS in this context may thus initiate a signalling cascade from NUAK1 through CAMKK2 to AMPK (Figure 2). Notably, direct phosphorylation of NRF2 by AMPK enhances NRF2-dependent transcription of specific target genes [60,61].

## NUAK substrates and signal transduction

Direct targets of NUAKs have been challenging to confirm, likely due to the strong association of either kinase with PP1β. A reasonable assumption is that NUAK substrate preference will be broadly similar to that of other ARKs, i.e. a strong preference for basic residues at positions -4 and -3, and small hydrophobic residues at positions -5 and +4, relative to the site of phosphorylation [62]. This appears to be borne out in kinome-wide phosphopeptide screening analysis [42]. By far the best characterised and most widely reproducible substrate of the NUAKs is the regulatory subunit of the Myosin phosphatase complex, MYPT1 (encoded by *PPP1R12A*), which is phosphorylated by either NUAK on serines 445, 472 and 910, resulting in 14-3-3 binding and attenuated phosphatase activity towards MYPT1-targeted phosphoproteins, including Myosin light chain (MYL) proteins [10]. Dynamic phosphorylation of MYL is required for cell motility, and loss of LKB1/NUAK1 enhances activity of the Myosin phosphatase complex, thereby promoting cell adhesion. Treatment of adherent cells with EDTA to promote detachment increases NUAK-dependent MYPT1 phosphorylation [10]. Accordingly, NUAK1 activation promotes cell migration, while NUAK1 inhibition strongly suppresses cell migration [45,49,63–65]. ROS also activate NUAK1 and acute treatment of cells with hydrogen peroxide strongly increases NUAK1-dependent MYPT1^S445^ phosphorylation [9]. ROS-mediated inactivation of PTEN rapidly activates AKT, with consequent inhibitory phosphorylation of GSK3β^S9^ [66]. Loss of NUAK1 thus enhances GSK3β^S9^ de-phosphorylation, with consequent suppression of NRF2 nuclear translocation, preventing the adaptative response to Oxidative stress and rendering cells hypersensitive to ROS [9].

**Figure 3:**
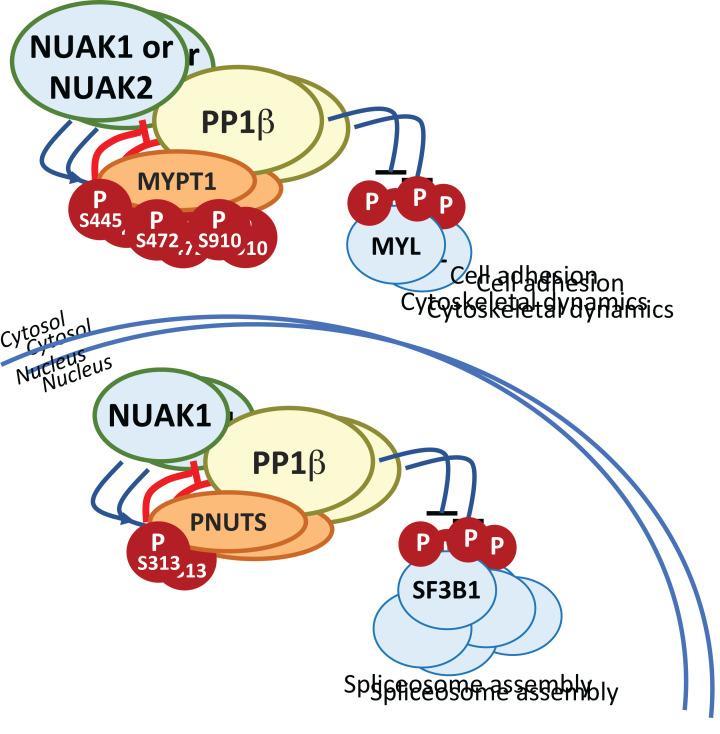
Regulation of PP1β complexes by NUAK Both NUAK1 and NUAK2 regulate cytosolic PP1β complexes via phosphorylation of the regulatory MYPT1 subunit. NUAK1 was additionally shown to regulate nuclear PP1β complexes via phosphorylation of a different regulatory subunit, PNUTS. It is currently unknown if this activity is shared with NUAK2.

NUAKs do not bind MYPT1 directly, but rather bind the catalytic subunit of the Myosin phosphatase, PP1β, via GILK motifs that are absent from other ARKs including AMPK – phosphorylation of MYPT1^S445^ is thus routinely used as a specific readout for NUAK activity [10]. Accordingly, NUAK1 was shown to phosphorylate a separate regulatory subunit of a nuclear PP1β complex, PNUTS, encoded by *PPP1R10*, on S313 [67]. As with MYPT1, phosphorylation of PNUTS attenuates PP1β-mediated phosphatase activity, this time towards the splicing complex subunit, SF3B1. Treatment of cells with either of 2 NUAK1-selective inhibitors blocked phosphorylation of PNUTS^S313^ and consequently that of SF3B1, suppressing formation of the spliceosome [67]. This raises the interesting possibility that NUAKs may regulate PP1β-containing phosphatase complexes more generally (Figure 3). Indeed, a number of additional PP1β-associated proteins have been identified in NUAK1-immunoprecipitated complexes [10,67]. The tight association and regulation of PP1β complexes by NUAKs presents a considerable challenge in disentangling potential direct targets of NUAK kinase activity from indirect effects of their restraint of PP1β phosphatase activity.

**Figure 4:**
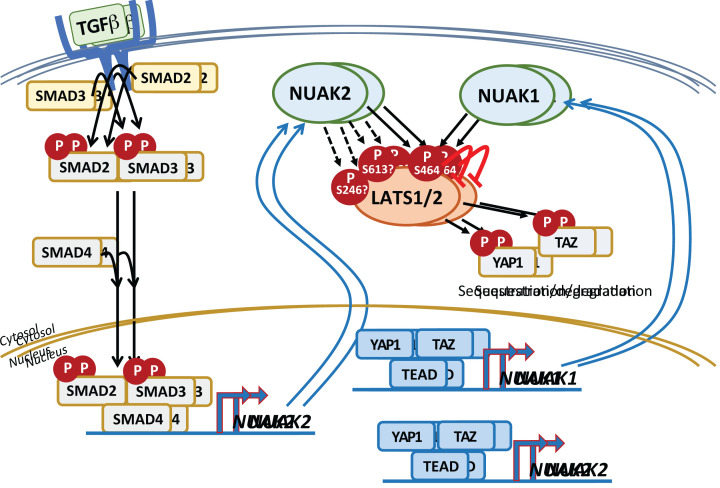
Integrated regulation of NUAK expression by TGFβ and HIPPO pathways Ligand- activated TGFβR phosphorylates SMAD2 & SMAD3, which then complex along with SMAD4 and translocate to the nucleus. SMAD2/3 complexes directly bind an enhancer on the *NUAK2* gene to drive expression. Both NUAKs inhibit HIPPO kinases LATS1/LATS2 by direct phosphorylation and potentially via inhibition of PP1β-dependent dephosphorylation, allowing unphosphorylated HIPPO effectors YAP1 and TAZ to translocate to the nucleus and activate TEAD-dependent gene expression. Expression of both *NUAK*s is directly regulated by YAP/TAZ/TEAD.

NUAK1 was found to regulate LATS1 stability during replicative senescence of diploid human fibroblasts [47]. Depletion of NUAK1 from WI-38 lung fibroblast cells prevented replicative senescence, whereas NUAK1 overexpression induced senescence prematurely. These effects were accompanied by LATS1 stabilization or reduction, respectively. Both NUAKs were able to phosphorylate a LATS1 peptide containing a putative ARK phospho-motif around S464, while S464A mutation rendered LATS1 refractory to NUAK overexpression [47]. As noted above, LATS1 is a key mediator of the HIPPO signalling cascade and directly targets YAP/TAZ to prevent their nuclear accumulation. As well as being a transcriptional target for YAP/TAZ, NUAK2 was shown to inhibit LATS1 kinase activity, and acute treatment of cancer cells with the dual NUAK inhibitors, WZ4003 or HTH-02-006, increased YAP1^S127^ phosphorylation and blocked nuclear translocation of YAP1 [21,22]. Although neither of these latter 2 studies confirmed NUAK-dependent phosphorylation of S464, mass spectrometric analysis of immunoprecipitated LATS1 following *in vitro* treatment with purified NUAK2 identified two additional putative phospho-sites, S246 and S613 [21]. Of the three potential sites, only S464 resides within a strong ARK motif and is predicted by the PP-PTMR to be phosphorylated by either NUAK [42]. All three studies however agree on an inhibitory role for NUAKs in regulating LATS, with a consequent feed-forward loop promoting oncogenic YAP/TAZ activity. Consistent with these findings, NUAK1 was recently shown to be induced in a YAP/TAZ-dependent manner during TGFβ-driven lung and liver fibrosis, and *Nuak1* floxed mice were protected from various models of injury-associated fibrosis [26]. NUAK1 overexpression reduced YAP1^S127^ phosphorylation and promoted nuclear accumulation of YAP1, similar to NUAK2, and this effect was retained in the presence of TGFβR kinase inhibitor. NUAKs thus appear to ‘lock-in’ YAP/TAZ-driven phenotypes following an initial TGFβ stimulation. Moreover, expression of *NUAK1* mRNA was strongly reduced in a familial case of anencephaly, caused by two recessive frameshift mutations in *NUAK2* [68]. Feed-forward regulation of YAP/TAZ via suppression of LATS1/2 by NUAK2 may thus be required to drive full expression of *NUAK1*, for instance during embryogenesis, accounting for the more severe phenotype of *Nuak2* deletion compared with *Nuak1* deletion *in utero* (Figure 4).

**Figure 5:**
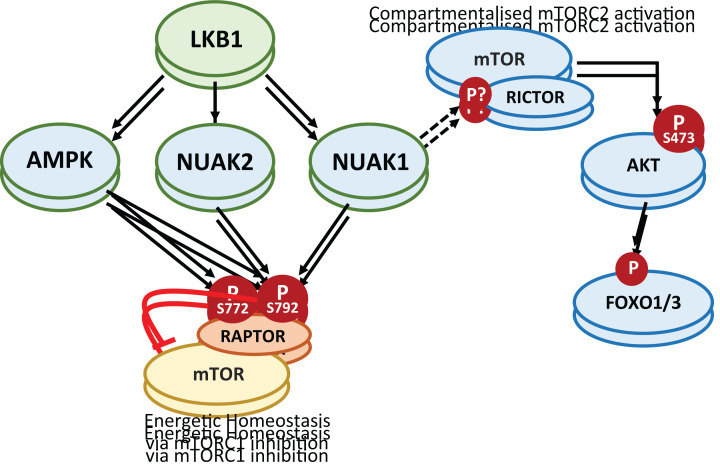
Regulation of mTOR complexes by NUAK Along with AMPK, NUAKs share the ability to restrict mTORC1 activity via inhibitory phosphorylation of the mTORC1 subunit, RAPTOR. NUAK1 was recently shown to also regulate mTORC2 subcellular localisation to promote activation of a specific pool of AKT, upstream of FOXO1/3 but not of TSC1/2. The mechanism of mTORC2 regulation is unknown but may relate to NUAKs role in cytoskeletal regulation via PP1β/MYPT1.

RNAi-based screening for synthetic lethal interactions with loss of *PTEN* in breast cancer identified *NUAK1*, along with *STK11* and *PIK3CB*, suggesting functional interaction between the LKB1-NUAK1 and PI3K-AKT pathways [69]. Mammalian target of rapamycin (mTOR) complexes are key participants in this pathway, with mTORC1 being a major effector of AKT signalling, while mTORC2 is required for full AKT activation, downstream of PI3K [70]. RAPTOR is a required component of the mTORC1 complex and is phosphorylated by AMPK on S722 and S792 to limit mTORC1 activity under conditions of energetic stress [71]. In *Prkaa1/a2* (encoding AMPKα1/α2) double deleted mouse embryo fibroblasts, residual phosphorylation of Raptor^S792^ is maintained by Nuak1 and Nuak2 [12]. Additionally, Rapamycin treatment rescued ATP homeostasis and cell viability in MYC overexpressing U2OS cells depleted of either NUAK1 or AMPKα1, indicating a functional requirement for NUAK in restraining mTORC1, similar to that of AMPK [8]. More recently, NUAK1 was shown to regulate mTORC2 sub-cellular localisation and activation of AKT [46]. Depletion or inhibition of NUAK1 altered the subcellular distribution of lysosomes, increased lysosomal accumulation of mTOR, and delayed mTORC2-dependent phosphorylation of AKT^S473^. The reduction of AKT^S473^ phosphorylation was observed in standard growth conditions, in the presence of oxidative stress and upon Insulin stimulation, and was accompanied by selective suppression of FOXO1/3a phosphorylation, but not of TSC2 phosphorylation, downstream of AKT (Figure 5). Although the same study suggested a degree of direct phosphorylation of AKT^S473^ by NUAK1, evidenced by increased phosphorylation *in vitro* with purified AKT and NUAK1, this site does not sit within a strong ARK motif and pronounced phosphorylation of the site in the absence of exogenous NUAK1 suggests the presence of a contaminating kinase or possible auto-phosphorylation [46].

A growing number of reports indicate NUAK1 regulates multiple aspects of mitochondrial biology. In developing neurons, NUAK1 traps mitochondria at presynaptic sites and sustains mitochondrial activity to promote formation of axonal branches: loss of NUAK1 results in diminished axonal ATP, shortened axons, and impaired axon branching [72–74]. This latter activity of NUAK1 was shown to depend on expression of a microprotein called BRAWNIN, loss of which phenocopies loss of NUAK1, and whose overexpression recued mitochondrial function and axon branching but not axonal length [74]. Whether regulation of BRAWNIN expression requires NUAK1 phosphorylation of PP1β^PNUTS^, or an alternative mechanism, is presently unknown. In cancer cells, NUAK1 overexpression enhances mitochondrial ATP production [58], while proteomic analysis following depletion of NUAK1 revealed reduced expression of multiple nuclear-encoded electron transport chain (ETC) proteins [8]. Acute inhibition of NUAK1 rapidly increases mitochondrial ROS production [8,9] and the reported reduction of ETC protein expression may thus reflect an adaptive response to elevated ROS [75]. No direct target of NUAK kinase activity has yet been linked to these phenotypes, although some aspects may be driven by regulation of PP1β and/or AKT/mTORC1 signalling by NUAK. NUAK1 was additionally shown to regulate mitochondrial dynamics, with elevated fusion observed in NUAK1-deleted Myeloma cells and more fragmented mitochondria evident in NUAK1-proficient cells [76]. NUAK1 deletion was associated with reduced S616 phosphorylation of the mitochondrial fission protein DRP1 along with elevated expression of Mitofusins, MFN1 and MFN2. The DRP1^S616^ site again does not resemble an ARK motif, however a putative consensus ARK motif is found at S637 and phosphorylation of DRP1^S637^ is enhanced by treatment with the metabolite AICAR with a concomitant reduction in DRP1^S616^ phosphorylation [77]. Intriguingly, AICAR was recently shown to activate NUAK1 in addition to its well-documented ability to activate AMPK [74]. DRP1^S637^ is thus a strong candidate for direct regulation by NUAK with functional implications affecting regulation of the mitochondrial network awaiting further investigation.

A final target of NUAK1 worth mentioning for its clinical relevance in Alzheimer’s disease is TAU (encoded by *MAPT*), independently found by two separate groups to be stabilised upon phosphorylation of S356 by NUAK1 [78,79]. The site corresponds to S673 of the isoform of TAU listed on PhosphoSite.org and is predicted by the PP-PTMR to be phosphorylated by all ARKs including NUAK1 and NUAK2. NUAK1 was found in TAU aggregates in Alzheimer’s disease post-mortem brain tissue, while genetic reduction or pharmacologic inhibition of NUAK1 reduced TAU phosphorylation, stability and accumulation in murine brains. Haploinsufficiency for NUAK1 moreover reduced symptoms in a mouse model of Tauopathy [78,79].

## Concluding remarks

Although often overshadowed by their more widely-studied cousins, the AMPK complexes, NUAKs are emerging to be important players in signal transduction, maintenance of redox and energetic homeostasis, alongside regulation of cell motility, with pathophysiological relevance for cancer and neurological diseases. Genetic evidence suggests non-redundant roles for either NUAK, whereas the confirmed molecular targets of NUAK kinase activity appear to be largely overlapping (e.g. MYPT1, RAPTOR, LATS1/2). It is possible that non-redundant roles emerge from differential cell/tissue-specific expression or alternative sub-cellular localisation, differences that may collapse in a disease setting. Additionally, NUAKs have both private (e.g. MYPT1) and overlapping (e.g. RAPTOR) targets with other ARKs, including AMPK. A number of NUAK-selective inhibitors are now available, some showing greater specificity than others, but the realisation of the therapeutic potential of such agents will require considerably deeper understanding of NUAKs’ biological activities. Pleiotropic effects arising from NUAK regulation of PP1β complexes, redundant and interwoven activities of the NUAKs, and overlap with AMPK (and likely other ARKs) in substrate regulation, each present significant challenges that will need to be overcome to achieve better understanding of NUAK in health and disease.

## Summary

- Structural/functional similarities between the NUAKs point to overlapping biological activities
- NUAKs are important regulators of PP1β protein phosphatase complexes
- NUAKs are key effectors of TGFβ and HIPPO pathways in cancer
- Emerging roles for NUAK in regulating mitochondrial health and activity

## Abbreviations

AKT: mammalian homologues of the *v-akt* oncogene
AMP: adenosine monophosphate
AMPK: AMP-activated protein kinase
ARK: AMPK-related kinase
CAMKK2: calcium/Calmodulin-dependent protein kinase kinase 2
DRP1: Dynamin-related protein 1
FOXO: Forkhead box transcription factor
GSK3β: gylcogen synthase kinase 3β
HIPPO: Hippopotamous like phenotype (*Drosophila melanogaster*)
LATS: large tumour suppressor
LKB1: liver kinase β1
MAPT: microtubule-associated protein Tau
miR: micro RNA
mTORC: mammalian target of Rapamycin complex
MYPT: myosin phosphatase targeting
NUAK: Nua (Gaelic translation of ‘new’) kinase
PKC: protein kinase C
PLK1: Polo-like kinase 1
PNUTS: phosphatase 1 nuclear targeting subunit
PP-PTMR: PhosphositePlus post-translational modification resource
PP1β: Protein phosphatase 1β
PTEN: Phosphatase and Tensin homologue
RAPTOR: regulatory associated protein of mTOR complex 1
ROS: reactive oxygen species
SMAD: small/mothers against decapentaplegic transcription factor
SNARK: sucrose non-fermenting AMPK-related kinase
STK: Serine/threonine kinase
TAZ: transcription coactivator with PDZ-binding motif
TEAD: TEA domain transcription factor
TGFβ: transforming growth factor β
TSC: Tuberous Sclerosis complex
YAP1: YES-associated protein 1
